# Unravelling the maternal evolutionary history of the African leopard (*Panthera pardus pardus*)

**DOI:** 10.7717/peerj.17018

**Published:** 2024-04-11

**Authors:** Declan R. Morris, Todd J. McWhorter, Wayne S. J. Boardman, Gregory Simpson, Jeanette Wentzel, Jannie Coetzee, Yoshan Moodley

**Affiliations:** 1 School of Animal and Veterinary Sciences, The University of Adelaide, Roseworthy, South Australia, Australia; 2 Department of Wildlife Studies, Faculty of Veterinary of Science, University of Pretoria, Onderstepoort, Gauteng, South Africa; 3 Department of Veterinary Tropical Diseases, Hans Hoheisen Wildlife Research Station, University of Pretoria, Onderstepoort, Gauteng, South Africa; 4 Mpumalanga Tourism and Parks Agency, Nelspruit, Mpumalanga, South Africa; 5 Department of Biological Sciences, University of Venda, Thohoyandou, Limpopo, South Africa

**Keywords:** Leopard, Mitochondrial DNA, NADH-5, South Africa, Evolutionary history, Population genetics, *Panthera pardus pardus*

## Abstract

The African leopard (*Panthera pardus pardus*) has lost a significant proportion of its historical range, notably in north-western Africa and South Africa. Recent studies have explored the genetic diversity and population structure of African leopards across the continent. A notable genetic observation is the presence of two divergent mitochondrial lineages, PAR-I and PAR-II. Both lineages appeared to be distributed widely, with PAR-II frequently found in southern Africa. Until now, no study has attempted to date the emergence of either lineage, assess haplotype distribution, or explore their evolutionary histories in any detail. To investigate these underappreciated questions, we compiled the largest and most geographically representative leopard data set of the mitochondrial NADH-5 gene to date. We combined samples (*n* = 33) collected in an altitudinal transect across the Mpumalanga province of South Africa, where two populations of leopard are known to be in genetic contact, with previously published sequences of African leopard (*n* = 211). We estimate that the maternal PAR-I and PAR-II lineages diverged approximately 0.7051 (0.4477–0.9632) million years ago (Ma). Through spatial and demographic analyses, we show that while PAR-I underwent a mid-Pleistocene population expansion resulting in several closely related haplotypes with little geographic structure across much of its range, PAR-II remained at constant size and may even have declined slightly in the last 0.1 Ma. The higher genetic drift experienced within PAR-II drove a greater degree of structure with little haplotype sharing and unique haplotypes in central Africa, the Cape, KwaZulu-Natal and the South African Highveld. The phylogeographic structure of PAR-II, with its increasing frequency southward and its exclusive occurrence in south-eastern South Africa, suggests that this lineage may have been isolated in South Africa during the mid-Pleistocene. This hypothesis is supported by historical changes in paleoclimate that promoted intense aridification around the Limpopo Basin between 1.0–0.6 Ma, potentially reducing gene flow and promoting genetic drift. Interestingly, we ascertained that the two nuclear DNA populations identified by a previous study as East and West Mpumalanga correspond to PAR-I and PAR-II, respectively, and that they have come into secondary contact in the Lowveld region of South Africa. Our results suggest a subdivision of African leopard mtDNA into two clades, with one occurring almost exclusively in South Africa, and we identify the potential environmental drivers of this observed structure. We caution that our results are based on a single mtDNA locus, but it nevertheless provides a hypothesis that can be further tested with a dense sample of nuclear DNA data, preferably whole genomes. If our interpretation holds true, it would provide the first genetic explanation for the smaller observed size of leopards at the southernmost end of their range in Africa.

## Introduction

Apart from humans, leopards (*Panthera pardus*) are possibly the most widely distributed and adaptable of all large mammals (Jacobson et al., 2016). The leopard is one of five species belonging to the genus *Panthera*, known collectively as big cats. Big cats are important in conservation biology as they are apex predators and influence trophic cascades (Ripple et al., 2014; Ford & Goheen, 2015). They are highly charismatic and generally attract large quantities of both tourism and conservation research funding and thus, also act as conservation umbrella species (Macdonald et al., 2015; Thornton et al., 2016; Albert, Luque & Courchamp, 2018). Genomic analyses suggest that the leopard is most closely related (sister taxa) to the lion (*P. leo*), and not the phenotypically similar jaguar (*P. onca*) (Figueiro et al., 2017). The same study also estimated that leopards and lions first diverged from a common ancestor approximately 2.57 million years ago (Ma) (95% CI [1.89–3.28] Ma) (Figueiro et al., 2017). This divergence time is also in line with fossil records, which estimate the emergence of both species occurred approximately two Ma (Werdelin & Sanders, 2010). The most basal leopard mtDNA clades and highest genetic diversity occur in Africa, suggesting an African origin for modern-day leopards with successful dispersal out of Africa into Europe and Asia occurring between 710 and 483 thousand (Ka) years ago (Uphyrkina et al., 2001; Paijmans et al., 2018, 2021).

Leopards once inhabited much of the Old World (Jacobson et al., 2016; Stein et al., 2016), and, given the scale and ecological heterogeneity of this distribution, have evolved into several taxonomically distinguishable groups (Miththapala, Seidensticker & Obrien, 1996; Uphyrkina et al., 2001). Although there have been up to 27 leopard subspecies described (including 12 subspecies on the African continent alone) the current taxonomic classifications recognise between eight (Miththapala, Seidensticker & Obrien, 1996) and nine subspecies (Uphyrkina et al., 2001). These are the African leopard (*P. p. pardus*), Arabian leopard (*P. p. nimr*), Persian leopard (*P. p. saxicolor*), Indian leopard (*P. p. fuscia*), Sri Lankan leopard *(P. p. kotiya*), Indochinese leopard (*P. p. delacouri*), North Chinese (*P. p. japonensis*), Siberian/Amur leopard (*P. p. orientalis*) and Javan leopard (*P. p. melas*). Miththapala, Seidensticker & Obrien (1996) did not recognise *P. p. nimr* as a subspecies however their study had minimal sample representation from the Arabian Peninsula compared with the number of samples from the rest of the world.

In Africa, the leopard was originally divided into two divergent phylogenetic lineages, Pardus lineage I (PAR-I) and Pardus lineage II (PAR-II), based on 611 base pairs (bp) of the mitochondrial NADH-5 gene and 116 bp of the mitochondrial control region (Uphyrkina et al., 2001). The divergence between these African lineages, both of which were contained within the same subspecies, was older than that between any other leopard subspecies lineage in the Eurasian part of the species range. Although their sample sizes were limited, Uphyrkina et al. (2001) noted that while PAR-I contained individuals from all across Africa, including southern Africa, PAR-II consisted of three individuals from South Africa (Kruger National Park) and one from Zimbabwe. Ropiquet et al. (2015) examined the same fragment of the NADH-5 gene plus 1,125 bp of the mitochondrial cytochrome *b* gene in 137 leopard samples from South Africa and neighbouring Mozambique, and also found significant support for two divergent maternal clades in southern Africa. All but one of their 35 Mozambique samples clustered into the first of these clades along with two individuals from the Kruger National Park, South Africa (KNP), while all other South Africa samples (Western Cape province, Eastern Cape province and Kwa-Zulu Natal province and the remainder of KNP) clustered into the second. Anco et al. (2018) produced NADH-5 sequences from an additional 56 specimens from museum archives (*n* = 41) and field samples (*n* = 15) from across Africa, and when analysed with the Ropiquet et al. (2015) dataset, they were able to separate African leopards into five regional clusters. PAR-I could now be further divided into four maternal clusters (West Africa, Coastal West-Central Africa, Central-East Africa and Central-Southern Africa) and PAR-II was renamed after the fifth cluster (Southern Africa). Interestingly, and despite a much-increased sample size, the composition of PAR-II still remained almost exclusively within South Africa, with only two of the 87 individuals belonging to PAR-II being from Mozambique. Also, in line with Uphyrkina et al. (2001), Anco et al. (2018) found that some individuals from South Africa (10/99) clustered outside PAR-II and into one of the minor clusters within PAR-I, that is, in their East-Central Africa and Central-Southern Africa clusters. However, it was not possible to determine the geographic locations for these ten PAR-I South Africa individuals from the data presented in that article. Finally, Morris et al. (2023) sequenced the NADH-5 and cytochrome *b* genes and generated 17 nuclear microsatellite profiles in a population genetic analysis of 33 leopards across a 700 m altitudinal gradient in the Mpumalanga province of north-eastern South Africa. When they analysed their mtDNA data set together with 16 publicly archived sequences from the adjacent Kruger National Park (Ropiquet et al., 2015), they observed the presence of two deeply divergent mtDNA lineages inhabiting the eastern and western parts of Mpumalanga, which corresponded to two nuclear genetic populations, connected by differing levels of bidirectional gene flow across South Africa’s Highveld-Lowveld divide. Since their sample set did not include leopards from elsewhere in Africa, they were unable to differentiate PAR-I from PAR-II, although previous studies show that individuals sampled south of Mpumalanga always clustered within PAR-II, and those from the north clustered almost always into PAR-I (Ropiquet et al., 2015; Anco et al., 2018). Taken together, these studies suggest that the northern part of South Africa may be the only region in Africa in which both PAR-I and PAR-II co-occur at high frequency. Paijmans et al. (2018) and Pecnerova et al. (2021) produced the first sets of complete leopard mitochondrial and nuclear genomes, respectively, however neither data set was representative enough in southern Africa to provide any further insights.

Therefore, despite previous observations of a major basal split in the maternal genetic structure of the African leopard (Uphyrkina et al., 2001; Ropiquet et al., 2015; Anco et al., 2018), determining haplotype distribution has been difficult, owing to sampling gaps and the use of differing regional sample sets (Uphyrkina et al., 2001; Ropiquet et al., 2015; Paijmans et al., 2018), and the loss of information when data sets are combined (Anco et al., 2018). Furthermore, the timing of the diversification of evolutionary clades has not been performed on the NADH-5 data, the only leopard locus for which a large sample from the entire species range is available. In addition, no demographic analysis of mitochondrial leopard populations has ever been conducted. Although mtDNA reflects only the maternal evolutionary history, its lower effective population size compared to nuclear DNA makes it more susceptible to genetic drift through time. Thus, past changes in female population size may be detectable through the use of demographic analyses of mtDNA alignments. Such analyses may provide a clearer picture of the drivers of genetic structure and, ultimately, provide for a better understanding of female evolutionary history.

To further investigate this aspect of African leopard evolutionary history, we compiled the most comprehensive NADH-5 data set to date by combining all homologous data from the studies mentioned above. This data set allowed not only a geographic interpretation of the data, but by applying molecular dating and demographic analyses we were able to time the divergences of both PAR-I and PAR-II, discuss their potential evolutionary drivers, and show that these divergent genetic groups are coming into secondary contact in the Mpumalanga province of South Africa.

## Methods

### Data set preparation

This study leverages mitochondrial NADH-5 data from all previous African leopard genetic studies to shed light on the evolutionary history of the leopard at the southernmost end of its range. Although the NADH-5 locus has been sequenced in 139 leopard specimens collected in South Africa, these data have never been analysed in combination with all the available data from the rest of Africa. Yet, such an analysis would be required to resolve the geographic distribution, and regions of potential overlap, of the ancient leopard clades PAR-I and PAR-II. This is particularly important, since the Morris et al. (2023) data set also represents the only leopard samples from the massive central plateau, known locally as the Highveld, and from along the entire eastward escarpment transition zone down to the Lowveld. This sample of 33 specimens collected in Mpumalanga, therefore, represents a number of ecologically diverse habitats in a region where two divergent maternal clades/nuclear populations have previously been found to overlap (Morris et al., 2023).

The genetic data set here assembled comprises the largest geographical sample for any African leopard study to date. These include 133 individual leopard specimens from Ropiquet et al. (2015) (South Africa and Mozambique), 56 from Anco et al. (2018) (samples sourced from all over Africa, comprised of a combination of museum specimens and samples collected from live animals by other researchers), 13 from Uphyrkina et al. (2001) (southern Africa–KNP, Botswana, Namibia), nine from Paijmans et al. (2018) (data from historical leopard museum specimens kept at the Natural History Museum of Denmark) and data for 33 specimens collected in Mpumalanga, sampled across the highveld-lowveld gradient of north-eastern South Africa (Morris et al., 2023) (Table 1). The accession numbers for the 33 NADH-5 leopard samples generated from the Mpumalanga province are OQ132962–OQ132992. Almost all studies provided an exact sample collection location, however the museum samples in Paijmans et al. (2018) and Uphyrkina et al. (2001) only specified a country and/or general region within a country of origin. We used a total of 244 NADH-5 sequences in this study, representing leopards from 18 countries across the African continent (Table 1). Consensus sequences for each individual were aligned in BioEdit (v7.0.5.3) (Hall, 1999) and checked for any errors, large gaps or missing data. The 244 samples in the final data set were trimmed to a length of 425 bp, corresponding to positions 12,632–13,058 in the complete mtDNA genome of *Felis catus* (Lopez, Cevario & Obrien, 1996) (Accession number U20753).

**Table 1:** A breakdown of all African leopard (*Panthera pardus pardus*) NADH-5 sequences used in this study.

Country	Specific locality	n =	Source
Algeria	N/A	1	Paijmans et al. (2018)
Angola	Chitau	1	Anco et al. (2018)
Botswana	Ngamiland, Bushman Pits	1	Anco et al. (2018)
	N/A	1	Uphyrkina et al. (2001)
Burundi	N/A	1	Paijmans et al. (2018)
Cameroon	N/A	13	Anco et al. (2018)
Chad	Fort Archambault District, N/A	2	Anco et al. (2018)
Democratic Republic of the Congo (D.R.C.)	Akenge, Faradje, Medje,	9	Anco et al. (2018)
	Gamangui, Ubangi District, Kivu		
	District		
	N/A	1	Paijmans et al. (2018)
Gabon	Lope NP	3	Anco et al. (2018)
Kenya	Cherangangi Hills, Elgeyo Forest	5	Anco et al. (2018)
	N/A	2	Paijmans et al. (2018)
Mozambique	N/A	1	Anco et al. (2018)
	Niassa Province	34	Ropiquet et al. (2015)
Namibia	Kaokoveld	1	Anco et al. (2018)
	N/A	3	Uphyrkina et al. (2001)
Nigeria	Gashaka-Gumti NP	1	Anco et al. (2018)
	N/A	1	Paijmans et al. (2018)
Republic of Congo	Domaine de Chasse de Mobko Hunting Reserve	1	Anco et al. (2018)
Senegal	Niokolo-Koba NP	10	Anco et al. (2018)
South Africa	Transvaal (ZA)	1	Anco et al. (2018)
	Western Cape (WC)	27	Ropiquet et al. (2015)
	Eastern Cape (EC)	11	Ropiquet et al. (2015)
	Kwa-Zulu Natal (KZN)	43	Ropiquet et al. (2015)
	Mpumalanga (LD, MY, LY, AN)	33	Morris et al. (2023)
	Kruger National Park (KNP)	18	Ropiquet et al. (2015)
	Kruger National Park (KNP)	5	Uphyrkina et al. (2001)
	N/A	1	Paijmans et al. (2018)
Tanzania	Rungwe, Serengeti Plains, Bamboo Forest	6	Anco et al. (2018)
	N/A	1	Paijmans et al. (2018)
Zambia	N/A	1	Anco et al. (2018)
	N/A	1	Paijmans et al. (2018)
Zimbabwe	N/A	4	Uphyrkina et al. (2001)
Total		244	

**Note:**

For the samples obtained in Mpumalanga Province, South Africa, locations are identified as; LD, Loskop Dam Nature Reserve; MY, Manyeleti Game Reserve; LY, Lydenburg area; AN, Andover Nature Reserve. One sample from South Africa was collected from the historical province known as Transvaal which now spans the modern-day Limpopo, Mpumalanga, Kruger National Park and Gauteng Provinces in South Africa. N/A denotes unknown region or location within country of origin.

### Genetic structure

To explore how mitochondrial DNA variation was structured and to date distinct clades of African leopard, phylogenetic trees were reconstructed from NADH-5 data using the program BEAST (Bayesian Evolutionary Analysis by Sampling Trees (v2.5.1)) (Bouckaert et al., 2019). A Bayesian framework allows the definition of parameters such as mutation rates, clock rates and ancestral calibration points as distributions and not simply point estimates. The substitution model (HKY + G) was selected by jModelTest (v2.1.10) (Darriba et al., 2012) using Bayesian Information Criterion (BIC). Heterogeneity in mutation rates was modelled using a gamma distribution with four bins. The clock model was defined by first running the analysis with relaxed lognormal and exponential clock options. The posterior distribution of the standard deviation of the clock rate did not include zero for the relaxed lognormal clock, suggesting the data fitted this model better than a strict clock. A relaxed lognormal clock rate of 0.0122 was set based on the known NADH-5 mutation rate in other feline species (Culver et al., 2000; Mukherjee et al., 2010).

Two nodes were calibrated with ancestral *Panthera* lineages. This necessitated the inclusion of the following outgroup taxa: the lion *(P. leo*, three sequences: accession numbers KP001498, KP001502 and KP001506) and three non-African leopard subspecies (*P. p. nimr*, *P. p. fuscia* and *P. p. orientalis*: Accession numbers AY035279, EF199743 and KY866876). Calibration priors were set up as follows: (1) the divergence between lion and leopard occurring at a mean of 2.57 Ma, with a normal distribution from 1.89 to 3.28 Ma (Figueiro et al., 2017) and (2) the divergence of Asian and African leopards at approximately 710 Ka with a normal distribution from 457–956 Ka. The analysis was run ten times independently with each run consisting of 200,000,000 Markov Chain Monte Carlo (MCMC) generations, sampling trees and parameters every 100,000 generations. Each run log was checked in Tracer (Rambaut et al., 2018). A burn in of 20% was discarded for each run. All post-burn in trees in all runs were combined using Logcombiner and a consensus tree was calculated using maximum clade credibility in Tree Annotator (both programs are in the BEAST package v2.5.1 (Bouckaert et al., 2019)). The above analysis was conducted separately on the full NADH-5 data set of 244 individual African leopards and then on the 47 leopard haplotypes that we observed in the data. Trees were edited in Figtree (v1.4.3) and Treegraph2 (v2.15.0) (Stover & Muller, 2010). Only nodes supported by a posterior probability of greater than 0.5 were shown in the haplotype tree, whereas the posterior probability was given for each node.

To investigate any non-tree-like evolution in African leopards, a median-joining haplotype network was reconstructed in Popart (v1.7) (Leigh & Bryant, 2015). Unlike a Bayesian analysis that can account for poor quality sequences, network reconstruction can be biased by samples with too much missing data. Therefore, sequences containing more than 5% missing data were excluded (28 samples from our dataset, see Table S1), resulting in the omission of five haplotypes compared to the haplotype tree.

### Mitochondrial phylogeography

Although some previous studies have shown that PAR-II occurred mainly in southern Africa (Uphyrkina et al., 2001; Anco et al., 2018), its exclusive occurrence only in the southern and south-eastern parts of South Africa and the increasing occurrence of PAR-I in northern South Africa suggests this country as the likely stronghold for PAR-II. We demonstrated this graphically by plotting haplotype distribution and frequencies onto the geographic space of the leopard’s range in Africa.

We further tested this “South Africa” hypothesis using a hierarchical analysis of molecular variance (AMOVA), analysing the haplotype frequencies of 16 hypothetical two-group partitions. These ranged from a scenario where only South Africa haplotypes comprised the first group, and all other African haplotypes the second group, to a hypothesis where all countries in southern Africa (South Africa, Namibia, Botswana, Zimbabwe and Namibia) comprised the first group. All combinations of southern African countries were tested, with South Africa always featuring in the first group. Variance components of the data and resulting F-statistics were tested for significance against a null hypothesis of zero variance using 10,000 permutations.

### Diversity

We estimated the genetic diversity of African leopard mitochondrial DNA clades using Arlequin (v3.5.2.2) (Excoffier & Lischer, 2010). Initially we performed the analysis with all the samples in the study (all of Africa) and then determined diversity indices for groups based on the two major clades determined from the phylogenetic tree (PAR-I and PAR-II lineages). To explore the diversity exclusive to leopards in South Africa, we also estimated diversity indices for the following two geographical groups: (1) South Africa and (2) the rest of Africa.

### Inferring historical demography

Despite the availability of many leopard mtDNA sequences, a demographic analysis has never been carried out. First, we tested our alignment against a null hypothesis of constant population size using Tajima (1989), Fu (1997) in Arlequin (v3.5.2.2). Positive values for Tajima’s D and Fu’s Fs can denote a past population contraction whereas negative values may indicate a population expansion. We also performed a mismatch distribution in Arlequin by calculating the sum of squared deviations (SSD) and raggedness. Significantly low SSD and raggedness can imply that a population underwent a sudden expansion (Rogers & Harpending, 1992).

We also modelled historical effective population size changes using a Bayesian Skyline model, which also leverages the demographic signal in DNA sequence alignments (Drummond et al., 2005). Here, we analysed PAR-I and PAR-II separately, to infer their independent demographic histories, although we concede that both clades contain significant structuring within them. The evolution of genetic structure can also lead to increases in historic effective population sizes, which can be mistaken for a population expansion on a Bayesian Skyline Plot (BSP). BSPs were reconstructed using BEAST and Tracer (Drummond et al., 2005; Bouckaert et al., 2019). Parameters were set using the same clock rate and models as the initial large phylogenetic tree however, each clade had a prior set based on the divergence time estimated for the corresponding node in the phylogenetic tree. MCMC was set at 50,000,000 permutations with a log every 10,000 retained. As with the phylogenetic analysis, MCMC convergence was determined in TRACER.

## Results

### Phylogenetic structure

**Figure 1:**
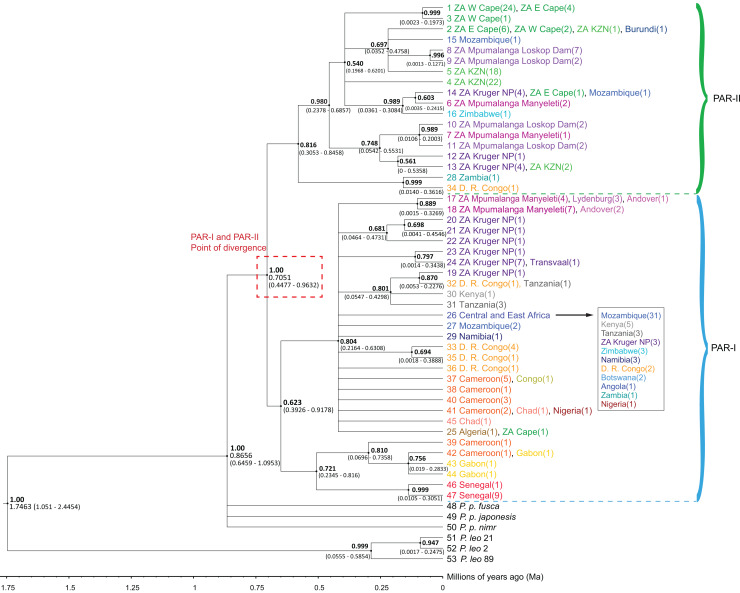
Phylogenetic tree of 47 African leopard (*Panthera pardus pardus*) NADH-5 haplotypes reconstructed using Bayesian inference in BEAST. The tree portrays the mitochondrial evolutionary history of the leopard across the African continent and highlights the relationship between the PAR-I and PAR-II mitochondrial lineages as the two oldest African leopard clades. Posterior probabilities are given in bold above each node (0.50–1.00). Nodes with less than 0.50 support were collapsed. Divergence times are given as a range of the 95% HPD underneath each supported node. Haplotypes are shaded based on region, with each country given unique colours. West Africa = red, East Africa = grey, Central Africa = yellow/orange, Southern Africa (Excluding South Africa) = blue, South Africa = green, and Mpumalanga province of South Africa = purple. The point at which PAR-I and PAR-II lineages diverged is highlighted in a red dashed box with median divergence time (0.7051) also given.

The phylogenetic trees (Figs. 1 and S1) highlighted an initial divergence of African leopard samples into PAR-I and PAR-II clades (posterior = 1). We dated this split to 0.7051 Ma (95% HPD [0.4477–0.9632] Ma) (Fig. 1). Both lineages then diversified at about the same time (PAR-I = 393–918 Ka, PAR-II = 305–846 Ka) giving rise to the observed diversity within each clade. PAR-I contained individuals from across Africa, however, only eight supported PAR-I clades contained more than three haplotypes, indicating that, apart from a major clade in western-central Africa (Cameroon and Gabon) and western Africa (Senegal), most haplotypes did not structure into clades with a clear geographic association (Fig. 1). PAR-II on the other hand, included 112 samples, with only five not originating in South Africa. These non-South Africa PAR-II individuals were sampled either in southern or central Africa (Zimbabwe, Mozambique, Zambia, DRC and Burundi). The two most basal PAR-II haplotypes were sampled in Zambia and the DRC, with the more derived PAR-II haplotypes in southern Africa, with the exception of one sample from Burundi with a haplotype identical to nine samples from South Africa. PAR-II was the only lineage sequenced in all leopards sampled in the Western Cape (*n* = 27), Eastern Cape (*n* = 11) and Kwa-Zulu Natal (*n* = 43) provinces of southern and south-eastern South Africa. The remainder of the South Africa PAR-II individuals were all from the north-eastern part of that country (Mpumalanga and the adjacent Kruger National Park), and these localities contained either PAR-I *(n* = 31) or PAR-II (*n* = 25), with the former more prevalent in the escarpment (Lydenburg, *n* = 3) and Lowveld (Manyeleti GR, *n* = 11; Andover NR, *n* = 3; Kruger National Park, *n* = 14) and the majority of the latter sampled on the Highveld (Loskop Dam, *n* = 13) and eight in the Lowveld (Manyeleti GR, *n* = 3; Kruger National Park, ncc = 9) (Figs. 1 and S1).

### Reticulate structure

**Figure 2:**
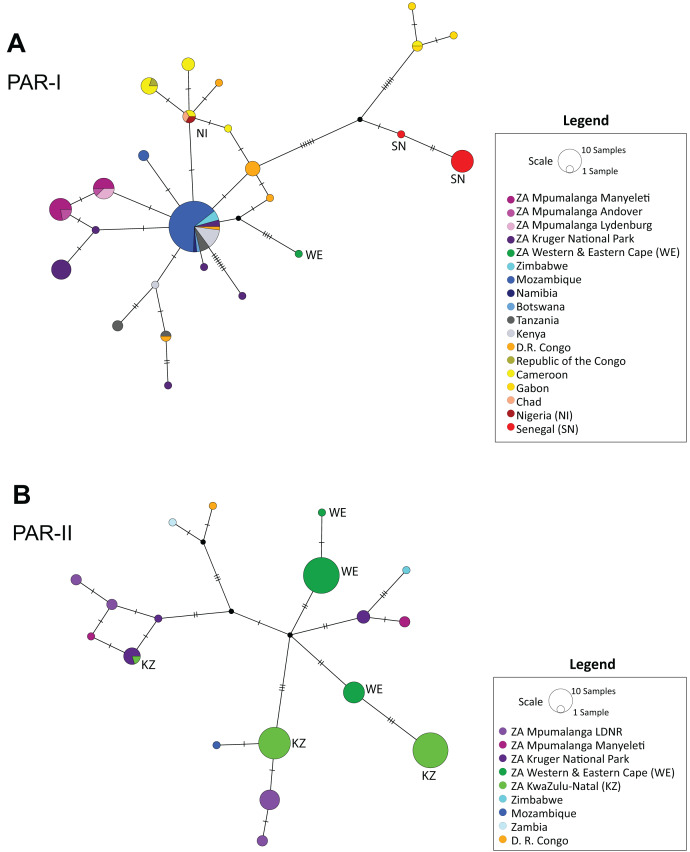
Median-joining haplotype networks showing the reticulate structure of African leopard (*Panthera pardus pardus*) NADH-5 haplotypes across the continent. (A) Network showing the structure of samples from the PAR-I mitochondrial lineage, and (B) Structure of the PAR-II mitochondrial lineage. In this figure, each country was uniquely colour coded. This was broken down further for samples collected from South Africa where all samples from the Mpumalanga province were assigned a differing shade of purple based on the reserve/region they were collected. LDNR = Loskop Dam Nature Reserve, Kruger National Park, Manyeleti GR, Andover NR and Lydenburg. Additional South African provinces were given a different shade of green (Western & Eastern Cape, KwaZulu-Natal). Five countries: South Africa, Zimbabwe, Mozambique, Zambia and Democratic Republic of the Congo (D. R. Congo) had both PAR-I and PAR-II haplotypes and are represented in both panels A and B. Countries and regions with a red or green colour have additional labelling to provide accessibility assistance for colour-blindness.

The median-joining haplotype network (Fig. 2) supported the structure observed in the phylogenetic tree (Figs. 1 and S1), confirming a single origin for the PAR-II lineage and showing that PAR-I and PAR-II were the first two lineages to diverge from the common maternal ancestor of all African leopards. Both tree and network also suggest that PAR-II is geographically structured internally, with unique haplotypes occurring in the Western-Eastern Cape, in KwaZulu-Natal, Mpumalanga and the Kruger National Park. PAR-I was also structured in western Africa, but less so in central and southern Africa with high levels of haplotype sharing among countries. PAR-I leopards in central and southern Africa appear to all belong to a phylogenetically diffuse set of haplotypes, all closely related to each other and with the most common haplotype (*n* = 55) centrally related to almost all others (Fig. 2).

### Spatial structure

**Figure 3:**
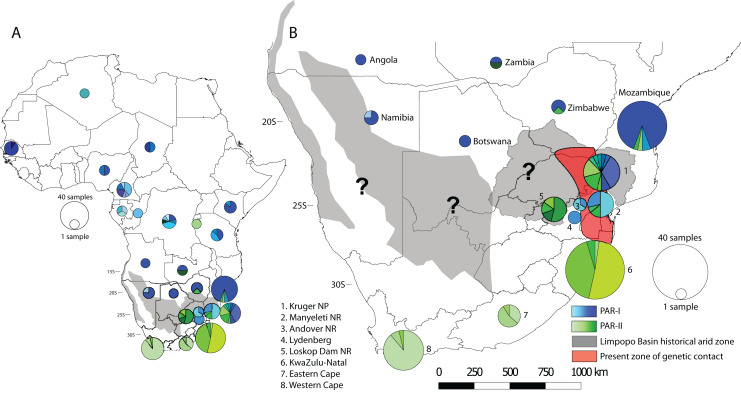
Spatial structure of PAR-I and PAR-II leopard lineages across Africa. PAR-I haplotypes (*n* = 29) are represented by differing shades of blue and PAR-II haplotypes (*n* = 18) are represented by differing shades of green. Pie graphs overlaid onto the map represent the number and frequency of haplotypes at that location. (A) The distribution of maternal haplotypes across the African continent. (B) The occurrence and overlap of PAR-I and PAR-II lineages in southern Africa. Grey shaded areas represent regions of intense aridity during the mid-Pleistocene, when gene flow may have been reduced. The red shaded region roughly encompassing the lower Limpopo basin and the Mpumalanga Lowveld suggests the region where PAR-I and PAR-II have come into present day secondary contact. Question marks denote other regions where PAR-I and PAR-II potentially overlap and may be coming into genetic contact. Base map of Africa sourced from ESRI: https://services8.arcgis.com/zNrTBuYXV2f35M0U/arcgis/rest/services/Africa_Countries/FeatureServer.

To visualise how maternal genetic variation was partitioned across geographic space, we plotted the distribution of haplotypes by location (Fig. 3). At a continental scale (Fig. 3A) PAR-I was distributed across most of the leopard’s African range from Algeria to northern South Africa. PAR-II, on the other hand occurs from the DRC and Zambia in Central Africa, with frequencies increasing in a southern direction. At the regional level of southern Africa (Fig. 3B), PAR-II haplotypes are at highest frequency in South Africa, occurring with PAR-I in the South African Lowveld (Kruger National Park and Manyeleti GR) and exclusively in leopards from the South African Highveld, KwaZulu-Natal and the Cape.

AMOVA analyses returned the highest possible F_CT_ (between group variation) and F_ST_ (variation among populations within groups) values for the two-group comparison separating PAR-I and PAR-II (Table 2). All F_ST_ values were significantly different from zero, although we concede that small sample sizes for some countries may have affected values at this hierarchical level. Furthermore, owing to a single degree of freedom in all other two-group scenarios, no F_CT_ value attained significance, although this statistic did vary appreciably (0.01956–0.11072, Table 2) reflecting the relative levels of variance attributable to each scenario. When several geographic scenarios partitioning different combinations of southern African countries with South Africa were tested, we found that the two-group scenario comparing South Africa with all other leopards maximised the values of both F-statistics, with the comparison including South Africa and Zimbabwe returning the second highest values. Interestingly, the above three grouping scenarios were all more likely than one of a single African leopard population (Table 2).

**Table 2:** Testing the “South African” hypothesis *via* AMOVA.

Group 1	Group2	F_ST_^*^	F_CT_^NS^
All African Leopards	n/a	0. 42226	n/a
South Africa (ZA)	All other countries	**0.43622**	**0.11072**
South Africa, Zimbabwe	All other countries	0.43606	0.10406
South Africa, Botswana	All other countries	0.43461	0.09559
South Africa, Namibia	All other countries	0.43319	0.08242
South Africa, Mozambique	All other countries	0.42879	0.02958
South Africa, Zimbabwe, Botswana	All other countries	0.43452	0.08994
South Africa, Zimbabwe, Namibia	All other countries	0.43315	0.07760
South Africa, Zimbabwe, Mozambique	All other countries	0.43107	0.03721
South Africa, Botswana, Namibia	All other countries	0.43165	0.06889
South Africa, Botswana, Mozambique	All other countries	0.42808	0.02544
South Africa, Namibia, Mozambique	All other countries	0.42767	0.02283
South Africa, Zimbabwe, Botswana, Mozambique	All other countries	0.43052	0.03369
South Africa, Zimbabwe, Botswana, Namibia	All other countries	0.43162	0.06477
South Africa, Zimbabwe, Namibia, Mozambique	All other countries	0.43034	0.03181
South Africa, Mozambique, Namibia, Botswana	All other countries	0.42706	0.01956
All Southern Africa (5 countries above)	All other countries	0.42997	0.02931
Pardus lineage 1 (PAR-I)	Pardus lineage 2 (PAR-II)	**0.64233**	**0.50894**

**Notes:**

**F**_ST_^*^, all F_ST_ vales were significant after permutation tests. **F**_**CT**_^**NS**^, apart from the last scenario (PAR-I *vs*. PAR-II, *p* < 0.0001), F_CT_ values were not significant owing to a single degree of freedom in two-group comparisons.

Analyses were run on multiple groupings of leopard populations in Africa, with combinations of all southern African countries tested together with South Africa. F_ST_, Variation among populations within groups, F_CT_, between group variations. Values in bold show the two data partitions with the highest levels of maternal structure, that is: South Africa *vs.* All other countries and PAR-I *vs.* PAR-II.

### Diversity and demography

Genetic diversity and demography indices were calculated for PAR-I, PAR-II and the whole data set. We also calculated the genetic diversity of all leopard samples collected from South Africa as we were interested to know what effect the existence of both PAR-I and PAR-II would have on the genetic diversity in that country (Table 3). A total of 47 haplotypes were observed among 244 African leopard NAHD-5 sequences (Table 3), and of these, 28 belonged to PAR-I and 18 to PAR-II. Haplotype diversity was high for both PAR-I (0.858) and PAR-II (0.895), but nucleotide diversity was higher in PAR-I. South Africa contained 34 haplotypes compared to the rest of the continent which displayed 36 different haplotypes (Table 3) and with levels of haplotype and nucleotide diversity exceeding either PAR-I and PAR-II, but slightly lower than the African total.

**Table 3:** Mitochondrial genetic diversity and demographic analyses of African leopard (*Panthera pardus pardus*) groupings.

Clade/Country	Name	n	Diversity				Demography			
			Polymorphic sites	Haplotypes	Haplotype diversity	Nucleotide diversity	Tajima’s D	Fu’s Fs	SSD	Raggedness
All leopards	Total	244	58	47	0.9363	0.0162	−0.8289	−24.5881**	0.0099	0.0163
Major lineages	PAR-I	132	43	29	0.8576	0.0089	−1.6206*	−25.6103***	0.0180*	0.0339*
	PAR-II	112	26	18	0.8945	0.0123	0.095	−7.8957*	0.0169	0.0332
South African leopards	South Africa	139	34	34	0.9226	0.0159	0.1983	−7.1485	0.0077	0.0167
All other African leopards	Africa-wide	105	44	36	0.8068	0.0103	−1.5359*	−20.0252***	0.0275	0.0455

**Note:**

n, number of samples in the group. Significance levels for results are indicated as:

* *p* < 0.05.

** *p* < 0.01.

*** *p* < 0.001.

When we tested these groups against a hypothesis of constant population size, both the total sample and PAR-I returned negative values for Tajima’s D and significantly negative values for Fu’s Fs, indicating a population expansion. On the other hand, only Fu’s Fs was significantly negative in PAR-II and Tajima’s D was positive. In the South Africa sample set, which contained both PAR-I and PAR-II, only Fu’s Fs was negative (Table 3). Similarly, mismatch distributions showed evidence for a population expansion only in the PAR-I lineage, with significant SSD and raggedness values (Table 3).

**Figure 4:**
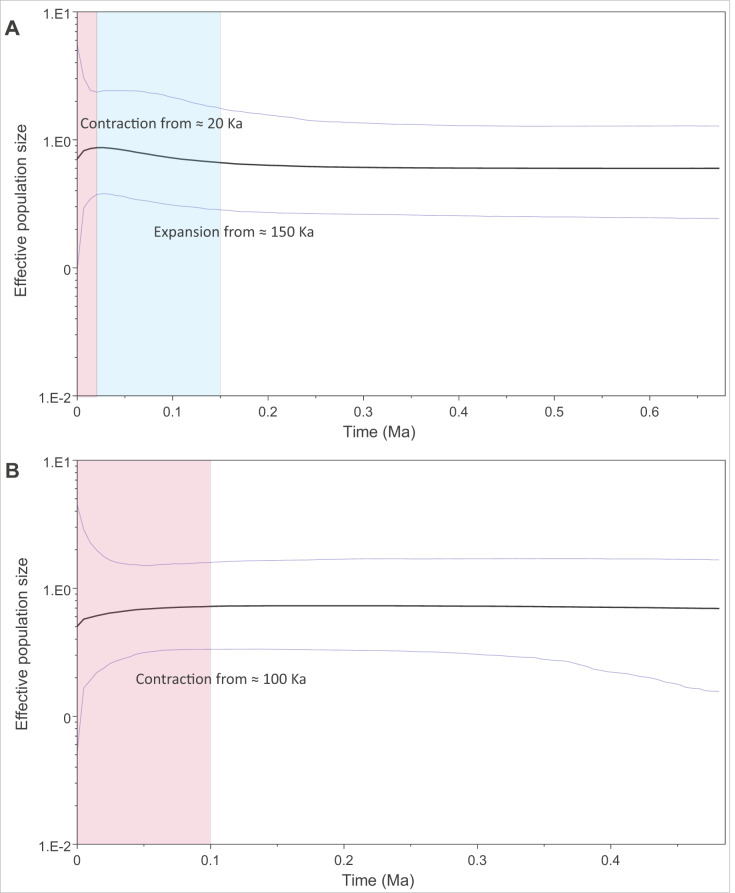
Changes in effective population size through the mid to late Pleistocene within the two major African leopard mitochondrial lineages (PAR-I and PAR-II). Demographic changes were modelled using a Bayesian skyline approach in BEAST. (A) Depicts PAR-I (*n* = 132) and (B) shows PAR-II (*n* = 112). Periods displaying a population expansion are highlighted in blue, and periods displaying a population contraction are highlighted in red.

We modelled past changes in effective population size using Bayesian skyline plots. Our results indicate that PAR-I underwent a population expansion beginning around 150 thousand years ago (Ka), followed by a more recent decline within the last 20 Ka (Fig. 4A). PAR-II, however, did not reflect a population expansion, but rather a steady contraction within the last 100 Ka to its lowest size today (Fig. 4B).

## Discussion

Despite several genetic studies on African leopards over the last two decades, the peculiar distributions of the major mtDNA Pardus lineages I and II have not been fully appreciated, and as a result, the potential evolutionary drivers of such structure have not been entirely explored. To shed more light on this major break in leopard mitochondrial genetic diversity we assembled the largest sample for which homologous genetic data were available.

### The distinctiveness of PAR-I and PAR-II

As in previous studies, we observed an initial divergence between the two African matrilineages PAR-I and PAR-II (Figs. 1 and 2). However, in this study we attempted to date the timing of splits using secondarily derived divergence estimates from a whole genome study on big cats (Figueiro et al., 2017). This analysis indicated a mid-Pleistocene split of an ancestral female African leopard lineage into the lineages PAR-I and PAR-II occurred between 447 and 963 Ka (Fig. 1). When we examined the data in geographic detail, it became clear that PAR-I did not extend to the southernmost leopard populations of the Cape and KwaZulu-Natal regions of South Africa, which were exclusively PAR-II. By analysing the Mpumalanga samples of Morris et al. (2023) in an African context, we show that their East Mpumalanga and West Mpumalanga clades were in fact none other than PAR-I and PAR-II, respectively. Intriguingly, Morris et al. (2023) also showed that East and West Mpumalanga were also differentiated at 17 microsatellite loci providing the tantalising prospect that PAR-I and PAR-II are also nuclear DNA populations. Other nuclear microsatellite studies did not highlight differences among African leopard samples (Uphyrkina et al., 2001; McManus et al., 2015; Ropiquet et al., 2015). In the case of Uphyrkina et al. (2001), only four individuals contained PAR-II haplotypes and none were from north-eastern South Africa where Morris et al. (2023) detected their major nuclear DNA discontinuity between Highveld and Lowveld samples. McManus et al. (2015) on the other hand, only studied populations within the exclusive range of PAR-II. Ropiquet et al. (2015) did not sample Highveld leopards, but nevertheless obtained a large South Africa data set, which included the Lowveld region and Mozambique which is around the southern limit of PAR-I. Although they claim that they did not find evidence for a nuclear population split across their sample set, it is clear in retrospect that the factorial correspondence analysis they conducted on their microsatellite data (Fig. 4 in Ropiquet et al. (2015)) revealed a major nuclear genetic discontinuity between samples mainly from Mozambique which formed a distinct cluster (PAR-I) that intercepted only with samples from the South African Lowveld (represented by the Kruger National Park) and not with samples from the Cape and KwaZulu-Natal (PAR-II). Thus, PAR-I and PAR-II are quite likely also nuclear DNA populations. Alternatively, distinct mtDNA patterns might also evolve if female leopards disperse to a lesser extent than males as has been suggested previously (Bailey, 1993; Fattebert et al., 2015). However, higher male dispersal may not translate into sex-biased gene flow as males tend to suffer higher natural mortality than females (Balme & Hunter, 2004; Daly et al., 2005). Population-scale sequencing of nuclear genomes sampled from southern Africa would help confirm or refute these interpretations.

### The leopards of South Africa

Our analyses of spatial structure showed that although PAR-II haplotypes can be found at a lower frequency in Central Africa, their frequency increases in south-eastern Africa (Zimbabwe, Mozambique and north-eastern South Africa), until they make up the only mtDNA lineage present at the southernmost extent of the species distribution (Fig. 3). AMOVA indicated that the highest variation between groups and among populations within groups was between South Africa and all other countries. Taken together the geographic distribution of haplotypes (Fig. 3) and the AMOVA results (Table 2) suggest strongly that the mtDNA lineage PAR-II, and possibly its associated nuclear DNA population, are typical of leopards of the South African Highveld and coastal regions from KwaZulu-Natal to the Cape of Good Hope. This part of South Africa is thus the stronghold for PAR-II diversity. The range of PAR-II up the western coast or in the Kalahari and Namib regions of north-western South Africa, southern Namibia and southern Botswana is yet to be determined (black question marks, Fig. 3B) and would require dense sampling in these areas.

### Evolutionary history of the African leopard

We used our data set to explore the maternal evolutionary history of leopards on the African continent. The divergence of PAR-I and PAR-II from a common ancestor occurred during the mid-Pleistocene transition (1,250–700 Ka) (Clark et al., 2006; Legrain, Parrenin & Capron, 2023), when glacial cycles increased from 40 to 100 ka, exposing sub-tropical regions to prolonged periods of aridity. As leopards are highly mobile and adaptable, it is unlikely that physical barriers such a mountains and rivers impede dispersal and gene flow over the long term. On the other hand, changes in the abundance and availability of resources (such as food, water and suitable habitat) are known to decrease leopard densities and, potentially, reduce gene flow (Hinde et al., 2023). Leopard populations that inhabit arid conditions (such as the Namib desert, the Kalahari and the Arabian Peninsula (Stuart, 1975; Bothma, 1998; Jacobson et al., 2016)) tend to exist in much lower densities. Densities of leopard in arid environments range from of 0.5 leopards per 100 km^2^ for central Namibia and 1.3–1.5 leopards per 100 km^2^ in the Kalahari. These are significantly lower than densities observed in other parts of Africa, which can range from 2.4 leopards per 100 km^2^ to 12.7 leopards per 100 km^2^ (Stander et al., 1997; Balme, Slotow & Hunter, 2010; Maputla, Chimimba & Ferreira, 2013; Strampelli et al., 2018; Morris et al., 2022). Leopard home ranges, which are largely governed by vegetation productivity, prey density and the density of other leopards (Santos et al., 2019; Snider et al., 2021; le Roex et al., 2022), are also much larger in arid regions. Therefore, while increased mid-Pleistocene aridity may not have prevented gene flow among African leopards, we suggest that it must have attenuated female gene flow enough to promote the divergence of PAR-I and PAR-II lineages.

The oldest split within PAR-I was in western Africa, but in the rest of Africa, PAR-I remained relatively undifferentiated throughout the late Pleistocene. Demographic analyses suggest that PAR-I underwent a population expansion (Table 3) beginning around 200 Ka (Fig. 4). The presence of one highly frequent haplotype that gave rise to all other non-West African PAR-I haplotypes is fully in line with this hypothesis. Under a population expansion, genetic drift is less likely to sort lineages across the landscape, explaining the lack of geographic structure among non-West African PAR-I leopards.

Unlike PAR-I, PAR-II was relatively well structured geographically, with much less haplotype sharing among locations and unique sets of haplotypes occurring in Central Africa, the Cape, KwaZulu-Natal and the South African Highveld (Loskop Dam Nature Reserve). Demographic analyses indicate that the effective population size of PAR-II was likely constant over the mid-Pleistocene with a gradual decline beginning around 100 Ka (Fig. 4). A major population contraction of PAR-II within southern or South Africa is unlikely from our data, especially in light of its high genetic diversity (Table 3). Nevertheless, higher drift relative to an expanding PAR-I is more likely to have led to the evolution of location-specific PAR-II haplotypes. Higher drift in PAR-II was also reflected in increased phylogenetic structure, with central African haplotypes (DRC and Zambia) diverging first, followed by the South African Lowveld, KwaZulu-Natal and finally the Eastern and Western Cape (Fig. 1). This phylogeographic structure suggests an origin for PAR-II in Central Africa and dispersal into southern Africa during the late Pleistocene.

The differing evolutionary trajectories of PAR-I and PAR-II indicate that they must have evolved largely in isolation from each other. As explained above, the only mechanism likely to attenuate gene flow in leopards is increased aridification and associated decreases in leopard population density. The aridification of the Limpopo Basin, at the junction of the southern African countries South Africa, Botswana, Zimbabwe and Mozambique (see Fig. 3), occurred approximately from 1.0 to 0.6 Ma (Caley et al., 2018) and could provide an explanation for the isolation of the more derived South African PAR-II haplotypes (95% HPD = 0.2378–0.6857 Ma) from leopards in other parts of Africa. This period of intense aridity was also significant for hominids in Africa, with the last of the Australopithecine ape men (*Paranthropus robustus*) disappearing from the fossil record around this time (Owen-Smith, 2021). A highly similar pattern of mtDNA genetic structure, also thought to be driven by mid-Pleistocene aridification, is apparent among Cape and grey-footed chacma baboons (*Papio ursinus*), with the unique haplotypes of both subspecies found only in northern South Africa (Sithaldeen, Ackermann & Bishop, 2015). Aridification in southern Africa is also suspected to have driven mtDNA structure in giraffe (*Giraffa cameloepardalis*, Brown et al. (2007)) and African ground squirrels (*Geosciurus inauris*, Herron, Waterman & Parkinson, 2005). Distinct South African mtDNA clades were also highlighted in lion (*Panthera leo*, Bertola et al. (2011)) and cheetah (*Acinonyx jubatus*, Charruau et al. (2011)) although these were not explicitly linked to Pleistocene aridification. Nevertheless, these distinct distributions of mtDNA diversity for a range of mammal species suggests genetic discontinuity in northern South Africa, potentially driven by an arid mid-Pleistocene environment.

Finally, the occurrence of PAR-I and PAR-II, beginning in north-eastern South Africa (Kruger National Park and Lowveld locations–Manyeleti GR and Andover NR) suggests that the two lineages must have come into recent secondary contact. Contemporary gene flow between PAR-I and PAR-II was also inferred from nuclear microsatellite data across the Highveld and Lowveld regions of Mpumalanga province (Morris et al., 2023). These results suggest that this part of South Africa is one of the regions of ongoing secondary contact between Africa’s two major African leopard populations. It may also be possible that PAR-I and PAR-II come into contact in the Kalahari and Namib regions (black question marks, Fig. 3B), however, low leopard population densities in these arid zones may work to attenuate gene flow and maintain the PAR-I/PAR-II divide. Only increased sampling in these regions, together with nuclear genome-scale data can help test these ideas.

### Sample provenance

While museum collections assembled during the 19^th^ and 20^th^ centuries provide an invaluable resource for evolutionary and phylogeographic studies, they are sometimes limited by incomplete or incorrect metadata. We suspect that two such specimens are contained within our data set. The first is ZMUC25, a leopard labelled “South Africa–Cape”, with no collection date. This sample has a PAR-I haplotype, which is the same as that of another sample from the same museum (ZMUC24) collected in Algeria in 1850. Given that Algeria and the Cape are on opposite ends of the continent, it is highly unlikely that both sampling localities are correct. Furthermore, during the 19^th^ and the first half of the 20^th^ century, the Cape was the only point of exit for all goods leaving South Africa, regardless of where in that vast country they originated. Therefore, given that PAR-I is not observed south of Mpumalanga province, it is unlikely that the label “the Cape” is accurate or precise enough. Similarly, the sample ZMUC3980, from the same museum again, but with the label “Burundi” and dated 1880, is almost certainly erroneous. This Burundi specimen has an identical haplotype to nine other leopards from South Africa, from the Cape and KwaZulu-Natal, in a region of the PAR-II tree that is exclusively South African. There is also the chance that the location data for both ZMUC25 and ZMUC3980 are correct, and that their non-unique NADH sequences were obtained through contamination in the laboratory.

## Conclusion

These results highlight South Africa, especially its southern and eastern regions comprising the Western Cape, Eastern Cape and KwaZulu-Natal, as the stronghold of one of Africa’s two main leopard genetic lineages. Although the findings presented here are limited to mtDNA, and the link between mtDNA lineages and nuclear genetic populations is speculative, further investigation using nuclear genome-scale data with greater sampling in southern Africa will help shed more light on our interpretations. Nevertheless, we are hopeful that our study will draw attention to an important and previously underappreciated aspect of African leopard evolution and biology. It has long been appreciated that African leopards at the southern end of their range were significantly smaller than their conspecifics in the Kruger National Park (Martins & Martins, 2006). The hypothesis we present here could provide a potential explanation for this difference in phenotype. Larger leopard sizes in the South African Highveld and KwaZulu-Natal, which are mainly PAR-II, could result from the ongoing secondary contact of both kinds of African leopard since the end of the Pleistocene, with a net influx of genes (and larger size) from the north and east to the south and west. Such a net influx of nuclear genes has already been demonstrated by Morris et al. (2023), where leopards are three times more likely to migrate from the Lowveld to the Highveld in Mpumalanga, hence with the potential to alter phenotypes in the Highveld rather than the Lowveld. The designation of a major leopard population inhabiting the southern and eastern parts of South Africa could place increased value on the leopards inhabiting this region.

## Supplemental Information

10.7717/peerj.17018/supp-1 Supplemental Information 1 Phylogenetic tree of 244 African leopard (*Panthera pardus pardus*) NADH-5 haplotypes reconstructed using Bayesian inference in BEAST. The tree portrays the mitochondrial evolutionary history of the leopard across the African continent and highlights the relationship between the PAR-I and PAR-II mitochondrial lineages as the two oldest African leopard clades. All numbers displayed on the nodes are the posterior probability of that node and is given as a number between 0.00–1.00, where 1.00 = 100% support that the associated clade exists. This tree acts as a supplemental figure to Figure 1 in this manuscript, where Figure 1 displays only haplotypes. Sample labels are shaded based on region, with each country given unique colours. West Africa = red, East Africa = grey, Central Africa = yellow/orange, Southern Africa (Excluding South Africa) = blue, South Africa = green, and Mpumalanga province of South Africa = purple.

10.7717/peerj.17018/supp-2 Supplemental Information 2 A breakdown of the 28 African leopard (*Panthera pardus pardus*) mitochondrial NADH-5 sequences that were removed from the haplotype spanning network analysis displayed in Figure 2. These samples were removed because they had greater than 5% of base pairs missing or unidentifiable. The sample ID, location and source are given.

10.7717/peerj.17018/supp-3 Supplemental Information 3 All African leopard mtDNA NADH-5 Sequences used in this study. All sequences were downloaded from GenBank.

## Additional Information and Declarations

### DNA Deposition

The following information was supplied regarding the deposition of DNA sequences: The Mpumalanga leopard samples are available at GenBank: OQ132962 to OQ132992.

### Data Availability

The following information was supplied regarding data availability: The NADH-5 mtDNA strands used in the BEAST analysis are available in the Supplemental File.
