# Supplementary material for: Unravelling the maternal evolutionary history of the African leopard (Panthera pardus pardus)
Source: PeerJ. 2024 Apr 11;12:e17018. doi: 10.7717/peerj.17018 (PMC11016244; doi:10.7717/peerj.17018)
Supplement: Supplemental Information 2 — These samples were removed because they had greater than 5% of base pairs missing or unidentifiable. The sample ID, location and source are given. [file peerj-12-17018-s002.docx]

| **Sample number removed** | **Sample ID** | **Location** | **Study Reference** |
| --- | --- | --- | --- |
| 1 | ZMUC5719NI | Nigeria | Paijmans et al. (2018) |
| 2 | ZMUC4446ZAM | Zambia | Paijmans et al. (2018) |
| 3 | ZMUC3980BRN | Burundi | Paijmans et al. (2018) |
| 4 | ZMUC24ALG | Algeria | Paijmans et al. (2018) |
| 5 | M81302TAN | Tanzania | Anco et al. (2018) |
| 6 | M80610ANG | Angola | Anco et al. (2018) |
| 7 | M52044DRC | Democratic Republic of the Congo | Anco et al. (2018) |
| 8 | M170305CAM | Cameroon | Anco et al. (2018) |
| 9 | M186944MZ | Mozambique | Anco et al. (2018) |
| 10 | M170309CAM | Cameroon | Anco et al. (2018) |
| 11 | M170301CAM | Cameroon | Anco et al. (2018) |
| 12 | M165802CHD | Chad | Anco et al. (2018) |
| 13 | leo7MZ | Mozambique | Ropiquet et al. (2015) |
| 14 | leo52WC | ZA_Western Cape | Ropiquet et al. (2015) |
| 15 | leo51WC | ZA_Western Cape | Ropiquet et al. (2015) |
| 16 | leo49WC | ZA_Western Cape | Ropiquet et al. (2015) |
| 17 | leo46WC | ZA_Western Cape | Ropiquet et al. (2015) |
| 18 | leo32WC | ZA_Western Cape | Ropiquet et al. (2015) |
| 19 | leo24EC | ZA_Eastern Cape | Ropiquet et al. (2015) |
| 20 | leo156KZN | ZA_KwaZulu-Natal | Ropiquet et al. (2015) |
| 21 | leo132KZN | ZA_KwaZulu-Natal | Ropiquet et al. (2015) |
| 22 | 83S2KNP | ZA_Kruger National Park | Uphyrkina et al. (2001) |
| 23 | 804S3BOT | Botswana | Uphyrkina et al. (2001) |
| 24 | 803S3NAM | Namibia | Uphyrkina et al. (2001) |
| 25 | 802S5NAM | Namibia | Uphyrkina et al. (2001) |
| 26 | 801S3NAM | Namibia | Uphyrkina et al. (2001) |
| 27 | 137S1KNP | ZA_Kruger National Park | Uphyrkina et al. (2001) |
| 28 | 136S8KNP | ZA_Kruger National Park | Uphyrkina et al. (2001) |

**Supplementary Table S1: A breakdown of the 28 African leopard (*Panthera pardus pardus*) mitochondrial NADH-5 sequences that were removed from the haplotype spanning network analysis displayed in Figure 2**. These samples were removed because they had greater than 5% of base pairs missing or unidentifiable. The sample ID, location and source are given.

**References**

Anco C, Kolokotronis SO, Henschel P, Cunningham SW, Amato G, Hekkala E (2018) Historical mitochondrial diversity in African leopards (*Panthera pardus*) revealed by archival museum specimens. *Mitochondrial DNA Part A* **29**:455-473. 10.1080/24701394.2017.1307973

Paijmans JLA, Barlow A, Forster DW, Henneberger K, Meyer M, Nickel B, Nagel D, Havmoller RW, Baryshnikov GF, Joger U, Rosendahl W, Hofreiter M (2018) Historical biogeography of the leopard (*Panthera pardus*) and its extinct Eurasian populations. *BMC Evol Biol* **18**:12. 10.1186/s12862-018-1268-0

Ropiquet A, Knight AT, Born C, Martins Q, Balme G, Kirkendall L, Hunter L, Senekal C, Matthee CA (2015) Implications of spatial genetic patterns for conserving African leopards. *C R Biol* **338**:728-737. 10.1016/j.crvi.2015.06.019

Uphyrkina O, Johnson WE, Quigley H, Miquelle D, Marker L, Bush M, O'Brien SJ (2001) Phylogenetics, genome diversity and origin of modern leopard, *Panthera pardus*. *Molecular Ecology* **10**:2617-2633. 10.1046/j.0962-1083.2001.01350.x
